# Procedures performed during neurosurgery residency in Europe

**DOI:** 10.1007/s00701-020-04513-4

**Published:** 2020-08-16

**Authors:** Martin N. Stienen, Christian F. Freyschlag, Karl Schaller, Torstein Meling, for the EANS Young Neurosurgeons and EANS Training Committee, Amir Al-Amin, Rafid Al-Mahfoudh, Aymeric Amelot, Lisa Arvidsson, Alkinoos Athanasiou, Cecilia I. A. Avellan, Luc Bauchet, Luka Berilazic, Ciaran Bolger, Pierre Bourdillon, Stathis Boviatsis, Oliver Bozinov, Pedro Branco, Werner Braunsdorf, Julian Cahill, Hans Clusmann, Jens Conrad, Dominik Cordier, Nuno Cristino, Djula Djilvesi, Johnny Duerinck, Chloé Dumot, Mehmet Akif Durak, Christian V. Eisenring, Giuseppe Esposito, Pierre-Jacques Finiels, Theofanis Flaskas, Christian F. Freyschlag, Stéphane Fuentes, Mario Ganau, Iordanis Georgiadis, Miltiadis Georgiopoulos, Dimitrios Giakoumettis, Nathalie Gilis, Catia Gradil, Stefan J. Grau, Andrey Grin, Georgios Hadjigeorgiou, Marc-Eric Halatsch, Nils Hecht, Markus Holling, Rosanda Ilic, Linda Iken, Nazaret Infante Santos, Timothee Jacquesson, Ibrahim Jalloh, Bojan Jelaca, Stefanie Kaestner, Darius Kalasauskas, Assylbek Kaliyev, Jean-Charles Kleiber, Juergen Konczalla, Karl F. Kothbauer, Vojin Kovacevic, Nenad Krajcinovic, Sandro M. Krieg, Olli-Pekka Kämäräinen, Mirko Lapcic, Christophe Lapras, Johan Ljungqvist, William B. Lo, Vincent Lubrano, Martin Majovsky, Romain Manet, Francesco Marchi, Yerkin Medetov, Torstein R. Meling, Ilaria Melloni, Anthony Melot, Patrick Mertens, Stephen Metcalfe, Svein H. Moerkve, Alexa Ruiz Mora, Erion Musabelliu, Mohammad J. Naushahi, Aitimbetov Nurzhan, Ibrahim Omerhodzic, Iddo Paldor, Johan Pallud, Vakis Papanastassiou, Vladimir Papic, Thanasis Paschalis, Michael Payer, Saskia M. Peerdeman, Philippe Peruzzi, Jenny Pettersson Segerlind, Jussi P. Posti, Francois Proust, Luca Regli, Jaako Rinne, Pierre-Hugues Roche, Saulius Rocka, Roman Rotermund, Scott A. Rutherford, Tönu Rätsep, Andreas Rüter, Ilkka M. Saarenpää, Mustafa Y. Samanci, Marko Samardzic, Nicolas Sampron, Ulrika Sandvik, Alba Scerrati, Karl Schaller, Michel Schneider, David B. Schul, Goksin Sengul, Emile Simon, Saurabh Sinha, Ole Solheim, Giorgio Spatola, Sergey Spektor, Martin N. Stienen, Jimmy Sundblom, Nikolaos C. Syrmos, Mario Teo, Simon Thomson, Nikolay Tonchev, Lazar Tosic, William P. Vandertop, Christian von der Brelie, Aleksic Vuk, James Walkden, Christopher Wendel, Mohammed Yaqout, Madina Yusupova, Gianluca Zollino

**Affiliations:** 1 Department of Neurosurgery, University Hospital Zürich, Zürich, Switzerland; 2 Clinical Neuroscience Center, University of Zürich, Frauenklinikstrasse 10, 8091 Zurich, Switzerland; 3 Department of Neurosurgery, Kantonsspital St.Gallen, St.Gallen, Switzerland; 4 Department of Neurosurgery, Medical University of Innsbruck, Innsbruck, Austria; 5 Department of Neurosurgery, Geneva University Hospitals, Geneva, Switzerland; 6 Faculty of Medicine, University of Geneva, Geneva, Switzerland

**Keywords:** Neurosurgery, Residency, Caseload, Europe, Training program, Working hour restriction

## Abstract

**Background:**

In a previous article (10.1007/s00701-019-03888-3), preliminary results of a survey, aiming to shed light on the number of surgical procedures performed and assisted during neurosurgery residency in Europe were reported. We here present the final results and extend the analyses.

**Methods:**

Board-certified neurosurgeons of European Association of Neurosurgical Societies (EANS) member countries were asked to review their residency case logs and participate in a 31-question electronic survey (SurveyMonkey Inc., San Mateo, CA). The responses received between April 25, 2018, and April 25, 2020, were considered. We excluded responses that were incomplete, from non-EANS member countries, or from respondents that have not yet completed their residency.

**Results:**

Of 430 responses, 168 were considered for analysis after checking in- and exclusion criteria. Survey responders had a mean age of 42.7 ± 8.8 years, and 88.8% were male. Responses mainly came from surgeons employed at university/teaching hospitals (85.1%) in Germany (22.0%), France (12.5%), the United Kingdom (UK; 8.3%), Switzerland (7.7%), and Greece (7.1%). Most responders graduated in the years between 2011 and 2019 (57.7%). Thirty-eight responders (22.6%) graduated before and 130 responders (77.4%) after the European WTD 2003/88/EC came into effect. The mean number of surgical procedures performed independently, supervised or assisted throughout residency was 540 (95% CI 424–657), 482 (95% CI 398–568), and 579 (95% CI 441–717), respectively. Detailed numbers for cranial, spinal, adult, and pediatric subgroups are presented in the article. There was an annual decrease of about 33 cases in total caseload between 1976 and 2019 (coeff. − 33, 95% CI − 62 to − 4, *p* = 0.025). Variables associated with lesser total caseload during residency were training abroad (1210 vs. 1747, *p* = 0.083) and female sex by trend (947 vs. 1671, *p* = 0.111), whereas case numbers were comparable across the EANS countries (*p* = 0.443).

**Conclusion:**

The final results of this survey largely confirm the previously reported numbers. They provide an opportunity for current trainees to compare their own case logs with. Again, we confirm a significant decline in surgical exposure during training between 1976 and 2019. In addition, the current analysis reveals that female sex and training abroad may be variables associated with lesser case numbers during residency.

**Electronic supplementary material:**

The online version of this article (10.1007/s00701-020-04513-4) contains supplementary material, which is available to authorized users.

## Introduction

The Young Neurosurgeons Committee and the Training Committee of the European Association of Neurosurgical Societies (EANS) are both committed to critically assess and improve training conditions in its member states and beyond. In a previous issue of *Acta Neurochirurgica*, we were given the opportunity to present some preliminary data from an EANS survey that aimed to determine the number of operative procedures conducted during neurosurgery residency in Europe [20]. As similar articles about the training conditions in Europe before [16–18], also this article stimulated some debate [5, 8, 19]. Some years back already, we had surveyed residents in training and asked them to indicate the average number of certain surgical procedures performed per month [16]. Albeit interesting, it remained unclear how representative the results were, since residents across all stages of training had been included. Therefore, to substantiate the prior work, we had now chosen a different approach, where we collected residency case logs from neurosurgeons who had already completed their training [16, 19, 20].

The preliminary findings were reported with the primary intentions to raise awareness for the survey, to attract more respondents, and to stimulate a debate about current training conditions of European residents in neurosurgery at the occasion of the EANS annual meeting 2019 in Dublin. We have been both encouraged and criticized for this work that—besides reporting mean case numbers—found an alarming decline in operative exposure over time [5, 8, 19].

The survey has remained open until recently and we here present the final results of our survey. Moreover, we evaluate, whether announcing preliminary survey results resulted in a significant change in responder characteristics or case numbers.

## Materials and methods

### Survey design and distribution

Details on the survey design and methods for distribution have been reported previously [20]. Ever since the publication of the preliminary results, further survey invitations were sent via email to EANS members once (October 2, 2019) and the survey link was advertised during the Young Neurosurgeon’s session at the EANS annual meeting in Dublin (Appendix). Again, no active social media platform advertisement was used, but neurosurgeons were permitted to share the survey link among their colleagues and peers. Multiple answers of the survey using the same IP address were impossible. No reminder emails were sent in case of non-response, to respect the decision of non-participation. Questionnaires of all responders between April 25, 2018, and until December 31, 2018, were basis of the preliminary result and all responses received until April 25, 2020, were included in this final analysis.

### Statistical considerations

To enable comparison with the prior report, we again illustrate results as mean and 95% confidence intervals (CI) for continuous variables or count and percent for categorical variables, respectively. *t* tests and chi-square tests serve for statistical comparisons. The report of caseloads and trends in time strictly follows the methodology applied previously, but is extended with regards to two aspects; first, we now additionally perform subgroup analyses for caseloads of those neurosurgeons having completed their training since implementation of the European Working Time Directive (WTD) 2003/88/EC (2005–2019). Second, we screen variables associated with residency caseloads by logistic regression and conduct in-depth analyses for identified variables indicative of trends (*p* < 0.2). Sensitivity analyses were also conducted, excluding results from Turkey since the total number of surgical procedures reported was found to be considerably higher in Turkey than for the rest of Europe (Supplemental Figure 1), while the reported actual length of residency training in Turkey (5.75 vs. 6.08 years, *p* = 0.609) was comparable.

Moreover, responder characteristics and caseload results are compared between the preliminary and the final cohort to determine whether the announcement of preliminary findings has led to any significant change.

The software used for the statistical analysis and graphical illustration was Stata v14.2 (StataCorp LP, College Station, TX, USA). *p* values < 0.05 were considered statistically significant.

### Ethical considerations

Survey participation was voluntary. No patient data was collected. Formal consent was not required for this type of study.

## Results

Until April 25, 2020, we received a total of 430 responses, of which 84 were excluded because responders indicated not having completed their training yet, 19 responses because participants had predominantly trained in non-European countries, and a further 159 responses due to incomplete and missing relevant data. Therefore, *n* = 168 responses were considered for the final analysis.

Details on the survey sample are summarized in Table 1. Survey responders had a mean age of 42.7 ± 8.8 years, and 88.8% were male. Responses mainly came from surgeons employed at university/teaching hospitals (85.1%) in Germany (22.0%), France (12.5%), the United Kingdom (UK; 8.3%), Switzerland (7.7%), and Greece (7.1%). Most responders graduated in the years between 2011 and 2019 (57.7%). Thirty-eight responders (22.6%) graduated before and 130 responders (77.4%) after the European WTD 2003/88/EC came into effect. The mean duration of residency was 6.1 ± 1.3 years.

**Table 1:** Basic demographic information of the survey responders. *SD* standard deviation

Age in years (mean ± SD)	42.7 ± 8.8
Gender	
Male Female	148 (88.1%) 20 (11.9%)
Type of hospital/employment	
University/teaching hospital Other public hospital Private hospital	143 (85.1%) 21 (12.5%) 4 (2.4%)
Country of training	
Albania Austria Belgium Bosnia and Herzegovina Croatia Czech Republic Estonia Finland France Germany Greece Israel Italy Kazakhstan Lithuania Netherlands Norway Poland Portugal Romania Russian Federation Serbia Sweden Switzerland Turkey UK	1 (0.6%) 2 (1.2%) 2 (1.2%) 1 (0.6%) 1 (0.6%) 1 (0.6%) 1 (1.3%) 8 (4.8%) 21 (12.5%) 37 (22.0%) 12 (7.1%) 2 (1.2%) 8 (4.8%) 3 (1.8%) 1 (0.6%) 4 (2.4%) 7 (4.2%) 1 (0.6%) 4 (2.4%) 1 (0.6%) 2 (1.2%) 9 (5.4%) 5 (3.0%) 13 (7.7%) 4 (2.4%) 14 (8.3%)
Length of residency (mean ± SD)	6.1 ± 1.3
Year of residency graduation	
1976–1980 1981–1985 1986–1990 1991–1995 1996–2000 2001–2005 2006–2010 2011–2015 2016–2019	2 (1.2%) 1 (0.6%) 5 (3.0%) 11 (6.5%) 9 (5.4%) 15 (8.9%) 28 (16.7%) 42 (25.0%) 55 (32.7%)
Part of training in a different country	
No Yes	122 (72.6%) 46 (27.4%)
Total	*n* = 168 (100%)

### Absolute numbers of procedures in general

The mean number of surgical procedures performed independently, supervised, or assisted throughout residency was 540 (95% CI 424–657), 482 (95% CI 398–568), and 579 (95% CI 441–717), respectively. Detailed numbers for cranial, spinal, adult, and pediatric subgroups are presented in Table 2.

**Table 2:** Overview on the caseloads of certain types of procedures, performed on average during neurosurgery residency in Europe. *CI* confidence interval

Procedure type	Independent	Supervised	Assisted	Total
	Mean, 95% CI	Mean, 95% CI	Mean, 95% CI	Mean, 95% CI
All procedures	540, 424–657	482, 398–568	579, 441–717	1594, 1318–1871
Cranial procedures	281, 225–337	257, 217–297	307, 239–375	850, 714–985
Spinal procedures	225, 160–291	219, 157–281	270, 191–348	717, 545–889
Procedures on adult patients	475, 380–570	431, 357–506	528, 400–656	1437, 1195–1678
Procedures on pediatric patients	49, 18–79	85, 11–160	62, 34–91	200, 101–301

In general, European trainees had more exposure to independently performed cranial as compared with spinal operations (281 ± 364 vs. 225 ± 426, *p* = 0.009). Exposure to cranial and spinal operations was similar for supervised (257 ± 249 vs. 219 ± 384, *p* = 0.079) and assisted procedures (307 ± 421 vs. 269 ± 487, *p* = 0.075).

Trainees were much more exposed to adult, as compared with pediatric procedures, including those performed independently (480 ± 612 vs. 49 ± 193, *p* < 0.001), supervised (431 ± 456 vs. 85 ± 462, *p* < 0.001), or assisted (531 ± 785 vs. 63 ± 175, *p* < 0.001).

### Absolute numbers of specific neurosurgical procedures

Table 3 summarizes mean numbers of specific neurosurgical interventions, again discriminated between those performed independently, supervised, or assisted throughout residency. In general, the simpler and less dangerous the procedure (e.g., burr hole trepanation, ventriculo-peritoneal (VP) shunt, peripheral nerve procedure, cranioplasty), the higher was the degree of independence, while assisted and supervised procedures were in general more complex and/or dangerous (e.g., infratentorial craniotomy, vascular procedure, trans-sphenoidal procedure, dorsal/lateral instrumented spine procedure; Table 3).

**Table 3:** Overview on the caseloads of specific types of procedures, performed on average during neurosurgery residency in Europe. *CI* confidence interval

Procedure type	Independent	Supervised	Assisted	Total
	Mean, 95% CI	Mean, 95% CI	Mean, 95% CI	Mean, 95% CI
Burr hole trepanation	137, 111–163	74, 35–113	39, 27–52	248, 176–320
Supratentorial craniotomy	131, 87–175	106, 87–125	130, 105–155	364, 288–441
Infratentorial craniotomy	23, 16–29	28, 32–34	48, 36–61	101, 78–123
Microsurgical treatment of vascular pathology	6, 2–11	13, 8–18	52, 38–65	71, 52–89
Endovascular procedure	1, 0–3	3, 1–4	6, 3–9	10, 5–14
Ventriculo-peritoneal shunt	48, 39–57	30, 24–35	33, 26–40	109, 93–125
Neuro-endoscopic procedure	5, 2–9	7, 5–8	10, 8–12	22, 16–27
Trans-sphenoidal procedure	7, 0–14	8, 5–10	25, 16–35	39, 25–53
Dorsal non-instrumented spine surgery	96, 62–131	83, 62–104	151, 105–197	331, 238–423
Anterior instrumented or non-instrumented spine surgery	30, 9–52	26, 19–33	51, 37–65	109, 70–148
Dorsal/lateral instrumented spine surgery	19, 0–40	22, 14–29	39, 21–57	80, 42–118
Cement augmentation	7, 0–15	4, 2–6	7, 4–11	18, 4–33
Functional procedure	21, 14–28	16, 12–20	28, 18–37	62, 46–79
Peripheral nerve procedure	20, 11–29	12, 8–16	16, 10–22	50, 33–66
Stereotactic radiosurgery	2, 0–4	3, 0–5	6, 2–11	11, 5–18
Cranioplasty	14, 11–17	12, 10–14	13, 10–16	39, 33–45

With an average of 137 procedures, burr hole trepanation was the procedure that was most frequently performed independently. Based on the responses of this survey, we are 95% confident that European residents perform between 111 and 163 such procedures independently throughout residency (Table 3). Other procedures performed relatively often were supratentorial craniotomies (average 131, 95% CI 87–175), dorsal non-instrumented spine procedures (average 96, 95% CI 62–131), and VP shunts (average 48, 95% CI 39–57). At the lower end of the spectrum, only few endovascular surgery (average 1, 95% CI 0–3), stereotactic radiosurgery (average 2, 95% CI 0–4), trans-sphenoidal surgery (average 7, 95% CI 0–15), or cement augmentation (average 7, 95% CI 0–15) procedure types were performed (Table 3).

The sensitivity analysis after exclusion of Turkish responses showed overall robustness of the models, while case numbers were slightly lower for certain procedure types (Supplemental Tables 1 and 2).

### Trend of residency caseload over time

**Fig. 1:**
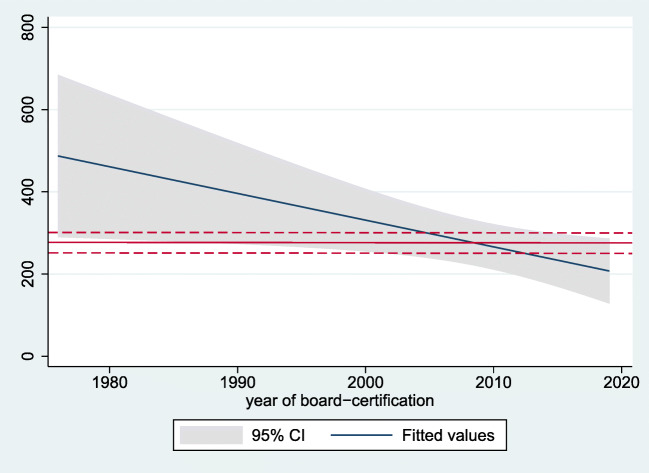
Linear prediction plot with 95% confidence intervals (CI), illustrating time trends (x-axis, year of residency graduation) in annual caseload (y-axis, number of procedures/year) for European neurosurgical residents. The fitted line indicates a decrease in caseload over time. In a linear regression model, there was an annual decrease of about 6 cases (coeff. − 6, 95% CI − 12 to − 1, *p* = 0.025). The red reference lines indicate the proposed threshold for adequate surgical training, ranging around 275 (250–300) per year and resident

We again analyzed whether the residency caseload changed over time. In a linear regression model, there was an annual decrease of about 33 cases in total caseload between 1976 and 2019 (coeff. − 33, 95% CI − 62 to − 4, *p* = 0.025). Also, the number of procedures performed per residency year (=case-year index) between 1976 and 2019 showed a decrease with about 6 cases less per year (coeff. − 6, 95% CI − 12 to − 1, *p* = 0.025). Figure 1 illustrates the decrease in mean annual caseload per resident over time, with the red lines indicating the suggested threshold for adequate training of 250–300 (mean 275) cases per year.

**Fig. 2:**
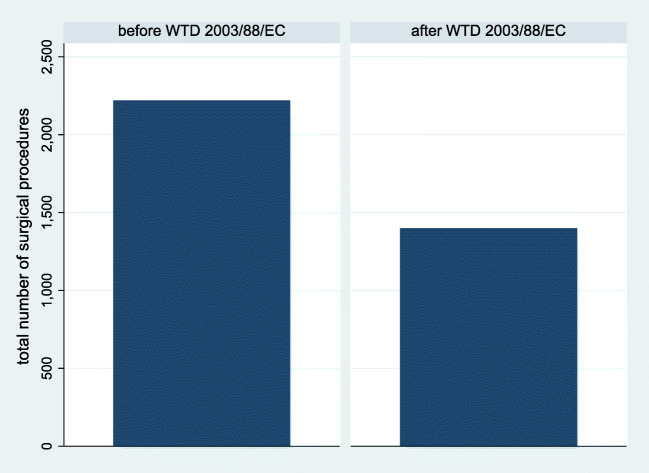
Bar chart illustration of the mean total number of surgical procedures (y-axis) performed throughout residency before and after introduction of the European Working Time Directive (WTD) 2003/88/ EC (x-axis). It was higher before as compared with after introduction of the WTD 2003/88/EC (2130 vs. 1338, *p* = 0.007).

When dichotomized for the time before or after introduction of the European WTD 2003/88/EC, the total residency caseloads (2219 ± 2768 vs. 1390 ± 1173, *p* = 0.012; Fig. 2) and procedures performed per residency year (=case-year index; 399 ± 580 (before) vs. 231 ± 192 (after), *p* = 0.008) were higher before as compared with after. Again, the difference between expected and observed caseload was positive before introduction of the European WTD 2003/88/EC but negative afterwards (576 ± 2829 vs. − 280 ± 1148, *p* = 0.009).

Again, all findings remained similar and statistically significant after exclusion of Turkish responses (data not shown).

### Variables associated with caseload

When screening for further variables associated with total caseload during residency (other than year of board certification) using a linear regression model, sex of the survey responder and training abroad were variables indicating trends, which is why subgroup analyses were conducted.

The total reported caseloads (both performed and assisted) during residency were 947 ± 527 for female and 1671 ± 1795 for male respondents (*p* = 0.111). The total caseloads performed and assisted by female residents during residency tended to be lower for cranial (462 ± 269 vs. 891 ± 857; *p* = 0.065), spinal (478 ± 357 vs. 742 ± 1095; *p* = 0.372), adult (823 ± 448 vs. 1504 ± 1517; *p* = 0.098), and pediatric (125 ± 145 vs. 208 ± 633; *p* = 0.639) cases, respectively.

The total reported caseloads (both performed and assisted) during residency were 1210 ± 874 and 1747 ± 1939 for trainees who did or did not perform parts of the residency in a different country (*p* = 0.083). The total caseloads for cranial (619 ± 381 vs. 939 ± 934; *p* = 0.036), spinal (513 ± 508 vs. 797 ± 1188; *p* = 0.141), adult (1065 ± 754 vs. 1581 ± 1636; *p* = 0.058), and pediatric (225 ± 857 vs. 191 ± 476; *p* = 0.759) cases were performed and assisted by trainees who did or did not perform parts of the residency in a different country.

There was no significant influence of the country of training on the total reported caseload (*p* = 0.443; Supplemental Figure 1).

### Preliminary vs. new cohort

The preliminary (*n* = 80) and the new cohort (*n* = 88) shared important responder features, including age, sex, type of hospital/employment, length of residency, year of residency graduation, and training abroad (all *p* > 0.05; Supplemental Table 3). There were statistically significant differences pertaining to the country of training (*p* < 0.001), however, with residents from countries like Germany, Greece, Italy, Netherlands, Portugal, and Turkey increasing and those from France, Norway, Sweden, Switzerland, and the UK decreasing their efforts (Supplemental Table 3).

In terms of caseload, the gross total caseload was similar for all, cranial, spinal, adult, and pediatric procedures (all *p* > 0.05; Supplemental Table 4). In subgroup analyses, caseloads of all procedures performed independently or supervised were similar in the preliminary and new cohort (all *p* > 0.05), but the number of assisted procedures was lower for all, cranial, spinal, and adult procedures (all *p* < 0.05; Supplemental Table 4), whereas it was similar for assisted pediatric procedures (*p* = 0.276).

## Discussion

The preliminary report of this survey [20] had already provoked some discussion about the training conditions for neurosurgeons in Europe, as an alarming decline in OR exposure was evident from the reported numbers [8]. However, the prior results had also been criticized by some for potentially being too high and not representative [5, 19]. The final survey results, contained in this article, were thus anticipated [5, 8, 19]. Here, the larger dataset serves as validation for the previous interim report.

As a first result, it is important to note that the responder-specific features of the current work (Table 1 and Supplemental Table 3) are remarkably comparable to the prior article [20], which indicates that the presentation (at the EANS 2019 annual meeting in Dublin) and publication of preliminary findings did not result in a significant change in the group of survey responders. Theoretically, the preliminary results could have triggered selective responses from people with an intention to drive the final results into a certain direction. However, this does not appear to be the case.

Second, the updated numbers are slightly corrected downward in general, as well as for both cranial and spinal procedures in adults. On the contrary, procedures in the pediatric population—both performed independently/supervised or assisted—remained stable or increased (Table 2 and Supplemental Table 2). As a result of the now 2.2 times greater sample size, the current report allows for a more accurate estimation of the actual training situation.

Regarding the quantity of specific procedures (Table 3), the results were overall quite comparable to the preliminary report, again with slightly lower mean case numbers for most types of procedures, except for microsurgical or endovascular treatment of vascular pathologies, ventriculo-peritoneal shunts, anterior and instrumented spine, and cement augmentation that were reported with higher case numbers. As the preliminary case numbers were criticized for potentially being somewhat too high and containing too little spine cases [5], the downward corrected final numbers with relatively more spine procedures may now be more realistic.

The previously reported trends—most importantly the decline in surgical cases during residency over time (Figs. 1 and 2)—could be reproduced with a lower effect size. While the annual decrease in caseload was 13 per year in the preliminary report, it was six cases per year in the final sample. This decline over time is likely a result of restricted working time in combination with an increase in the administrative burden for today’s resident physicians [13, 18], as well as increase in the number of trainees per department, change in surgical indications and decline of total cases per department over time [20]. Altogether, it can be stated that we did not observe a major change, but a certain adjustment in the previously reported results after having increased the sample size. It remains unclear from our data, whether or not the decline in caseload during training translates into worse performance as a neurosurgeon, higher complication rates, or worse postoperative outcomes. Supervised surgical training does not increase complication rates or worsens outcome, as could be shown before [9–11, 21, 22]. However, potentially dangerous surgery performed by unexperienced surgeons without adequate supervision is unsafe and data from the USA shows an increase in complication rates and morbidity after caseloads were reduced by duty-hour restrictions [2, 3, 7]. The progressive use and increasing quality of both teaching didactics and technology (e.g. neuronavigation, intraoperative magnetic resonance imaging, simulation, virtual reality, or preoperative case-specific 3D-printing) and additional means for training (e.g. EANS training courses) may be able to partially compensate for the declining case numbers [1, 4, 6, 20, 24]. Further research is needed to delineate how many surgical cases are at a minimum required during training in order to safely perform surgery independently. Where is the cutoff between “dangerous” and “safe” to be set? This might be a threshold that varies between countries or regions. The declining caseloads have some important implications on the future of European neurosurgery training (earlier hands-on practice vs. longer residency vs. introduction of additional fellowship training) that are discussed at length in our preliminary report and remain valid with the updated results [20].

The higher number of responses with the final cohort allowed us to conduct more in-depth analyses, e.g., pertaining to the relationship between the trainee’s gender and caseload. For general surgery, it is known that female surgeons provide exceptional care with complication rates and outcomes that are similar or even slightly superior compared with their male counterparts [14, 23]. We did notice a tendency for female residents in Europe to perform less cases during training. Although the relationship between caseload and gender was statistically not significant, it was evident for all major subgroups, incl. cranial, spinal, adult, and pediatric. With only 20 female survey responders, many of which (95%) completed their training after introduction of the European WTD 2003/88/EC; the robustness of the finding is questionable, though important to mention. The number of female neurosurgical trainees in Europe has lately increased with < 20% female participation at the EANS training courses in 2009 and a number that now approaches 25% [15]. Previous research about neurosurgery training where we surveyed residents had a 24% participation of females, whereas this rate was halved in the current article for which we surveyed board-certified neurosurgeons (Table 1). There is no robust data available to estimate the male-female ratio in Europe (and evaluate, whether the male-female ratio among our responders is representative), but it can be assumed that—similar to the situation found in the USA and Canada—females are likely still underrepresented in academic neurosurgery [12]. This area of research deserves to be further intensified. Our current findings at least call for close observation of the quality of training with regard to the trainee’s gender.

We were first surprised to find that “training abroad” was a variable associated with lower caseload during residency since trainees usually opt to rotate into specialized department internationally in order to gain operative exposure and hereby increase their personal surgical experience. However, in the way how the question was designed, other forms of “away rotation”—e.g., for research purpose—may also have been included under this category.

### Strengths and limitations

The strength of this survey-based study is its high representativeness, having succeeded to obtain responded from neurosurgeons working in 26 European countries. Furthermore, the objectivity and precision of the results should be high, considering that participants were asked to respond based on their actual case logs from residency, whenever available. Weaknesses remain, including the possibility of selection and recall bias or the lack of a true control with regards to the accuracy of case logs, but those have been discussed extensively before [5, 19, 20]. Also, despite a long period where the survey was active, the participant number remains low and some countries are only represented by single or few participants. Accordingly, the generalizability of the final case numbers—as collected with this survey—must be interpreted cautiously.

## Conclusion

In summary, the updated and now final results from this EANS survey on training conditions in European residency only modify the previous findings to a minor extent. Those numbers serve as a reference frame for individual trainees to compare with, in times where no better data is available. Again, we found a significant decrease in operating room exposure during neurosurgery residency over the last decades, which is likely a result of less working time and distribution of the caseload among more trainees, as well as parallel increase in administration and bureaucracy. In addition, the current analysis reveals that female sex and training abroad may be variables associated with lesser case numbers during residency, whereas caseloads appear comparable across different EANS member countries.

## Electronic supplementary material

ESM 1 (PDF 24 kb)

ESM 2 (PDF 35 kb)

ESM 3 (PDF 26 kb)

ESM 4 (PDF 27 kb)

Supplementary Figure 1 Box plots of the total number of surgical procedures performed and assisted during residency by trainees from different EANS countries. The figures display the median with the 25th–75th percentile (box), the upper and lower adjacent values (whiskers). Outliers are not visualized. (PDF 18 kb)

## Appendix

* Contributors of the “*EANS YNC and TC*” (listed in alphabetical order):

- Amir Al-Amin
- Rafid Al-Mahfoudh
- Aymeric Amelot
- Lisa Arvidsson
- Alkinoos Athanasiou
- Cecilia I. A. Avellan
- Luc Bauchet
- Luka Berilazic
- Ciaran Bolger
- Pierre Bourdillon
- Stathis Boviatsis
- Oliver Bozinov
- Pedro Branco
- Werner Braunsdorf
- Julian Cahill
- Hans Clusmann
- Jens Conrad
- Dominik Cordier
- Nuno Cristino
- Djula Djilvesi
- Johnny Duerinck
- Chloé Dumot
- Mehmet Akif Durak
- Christian V. Eisenring
- Giuseppe Esposito
- Pierre-Jacques Finiels
- Theofanis Flaskas
- Christian F. Freyschlag
- Stéphane Fuentes
- Mario Ganau
- Iordanis Georgiadis
- Miltiadis Georgiopoulos
- Dimitrios Giakoumettis
- Nathalie Gilis
- Catia Gradil
- Stefan J. Grau
- Andrey Grin
- Georgios Hadjigeorgiou
- Marc-Eric Halatsch
- Nils Hecht
- Markus Holling
- Rosanda Ilic
- Linda Iken
- Nazaret Infante Santos
- Timothee Jacquesson
- Ibrahim Jalloh
- Bojan Jelaca
- Stefanie Kaestner
- Darius Kalasauskas
- Assylbek Kaliyev
- Jean-Charles Kleiber
- Juergen Konczalla
- Karl F. Kothbauer
- Vojin Kovacevic
- Nenad Krajcinovic
- Sandro M. Krieg
- Olli-Pekka Kämäräinen
- Mirko Lapcic
- Christophe Lapras
- Johan Ljungqvist
- William B. Lo
- Vincent Lubrano
- Martin Majovsky
- Romain Manet
- Francesco Marchi
- Yerkin Medetov
- Torstein R. Meling
- Ilaria Melloni
- Anthony Melot
- Patrick Mertens
- Stephen Metcalfe
- Svein H. Moerkve
- Alexa Ruiz Mora
- Erion Musabelliu
- Mohammad J. Naushahi
- Aitimbetov Nurzhan
- Ibrahim Omerhodzic
- Iddo Paldor
- Johan Pallud
- Vakis Papanastassiou
- Vladimir Papic
- Thanasis Paschalis
- Michael Payer
- Saskia M. Peerdeman
- Philippe Peruzzi
- Jenny Pettersson Segerlind
- Jussi P. Posti
- Francois Proust
- Luca Regli
- Jaako Rinne
- Pierre-Hugues Roche
- Saulius Rocka
- Roman Rotermund
- Scott A. Rutherford
- Tönu Rätsep
- Andreas Rüter
- Ilkka M. Saarenpää
- Mustafa Y. Samanci
- Marko Samardzic
- Nicolas Sampron
- Ulrika Sandvik
- Alba Scerrati
- Karl Schaller
- Michel Schneider
- David B. Schul
- Goksin Sengul
- Emile Simon
- Saurabh Sinha
- Ole Solheim
- Giorgio Spatola
- Sergey Spektor
- Martin N. Stienen
- Jimmy Sundblom
- Nikolaos C. Syrmos
- Mario Teo
- Simon Thomson
- Nikolay Tonchev
- Lazar Tosic
- William P. Vandertop
- Christian von der Brelie
- Aleksic Vuk
- James Walkden
- Christopher Wendel
- Mohammed Yaqout
- Madina Yusupova
- Gianluca Zollino

## Abbreviations

CA: California
CI: Confidence interval
EANS: European Association of Neurosurgical Societies
TX: Texas
UK: United Kingdom
USA: United States of America
VP: Ventriculo-peritoneal
WTD: Working time directive
