# Supplementary material for: Procedures performed during neurosurgery residency in Europe
Source: Acta Neurochir (Wien). 2020 Aug 16;162(10):2303–11. doi: 10.1007/s00701-020-04513-4 (PMC7496021; doi:10.1007/s00701-020-04513-4)
Supplement: Supplementary file 1 — (PDF 24 kb) [file 701_2020_4513_MOESM1_ESM.pdf]

Supplemental Table 1: Overview on the caseloads of certain types of procedures, performed on average during neurosurgery residency in Europe after excluding survey responses from Turkey. CI = confidence interval.

**Procedure type**	**Independent**	**Supervised**	**Assisted**	**Total**
	Mean, 95% CI	*Mean, 95% CI*	*Mean, 95% CI*	*Mean, 95% CI*
All procedures	479, 410 – 548	463, 382 – 545	573, 433 – 714	1500, 1257 – 1743
Cranial procedures	251, 216 – 286	246, 209 – 282	300, 232 – 368	796, 684 – 909
Spinal procedures	193, 153 – 232	211, 150 – 272	270, 190 – 351	673, 514 – 832
Procedures on adult patients	428, 364 – 492	419, 346 – 492	525, 394 – 656	1369, 1147 – 1590
Procedures on pediatric patients	31, 22 – 40	79, 3 – 155	59, 30 – 88	172, 80 – 264
