# Supplementary material for: Procedures performed during neurosurgery residency in Europe
Source: Acta Neurochir (Wien). 2020 Aug 16;162(10):2303–11. doi: 10.1007/s00701-020-04513-4 (PMC7496021; doi:10.1007/s00701-020-04513-4)
Supplement: Supplementary file 2 — (PDF 35 kb) [file 701_2020_4513_MOESM2_ESM.pdf]

Supplemental Table 2: Overview on the caseloads of specific types of procedures, performed on average during neurosurgery residency in Europe after excluding survey responses from Turkey. CI = confidence interval.

Procedure type	Independent	Supervised	Assisted	Total
	Mean, 95% CI	*Mean, 95% CI*	*Mean, 95% CI*	*Mean, 95% CI*
Burr hole trepanation	133, 107 – 159	71, 32 – 110	35, 25 – 45	235, 165 – 306
Supratentorial craniotomy	112, 89 – 135	102, 84 – 120	126, 102 – 151	335, 282 – 388
Infratentorial craniotomy	21, 15 – 27	26, 20 – 32	47, 35 – 60	96, 74 – 117
Microsurgical treatment of vascular pathology	5, 2 – 8	11, 7 – 15	51, 38 – 64	67, 50 – 83
Endovascular procedure	1, 0 – 3	3, 1 – 4	6, 3 – 9	10, 5 – 14
Ventriculo-peritoneal shunt	48, 38 – 57	30, 24 – 34	32, 25 – 38	107, 91 – 123
Neuro-endoscopic procedure	4, 2 – 5	6, 5 – 8	10, 8 – 12	20, 16 – 25
Trans-sphenoidal procedure	4, 1 – 6	8, 5 – 10	25, 16 – 35	35, 24 – 47
Dorsal non-instrumented spine surgery	94, 59 – 129	81, 60 – 103	149, 103 – 196	325, 231 – 419
Anterior instrumented or non-instrumented spine surgery	30, 9 – 52	25, 19 – 32	50, 37 – 64	107, 67 – 147
Dorsal/lateral instrumented spine surgery	19, 0 – 40	22, 14 – 30	38, 20 – 56	78, 40 – 117
Cement augmentation	6, 0 – 15	4, 2 – 6	7, 3 – 11	18, 4 – 33
Functional procedure	21, 14 – 28	17, 13 – 21	28, 18 – 37	63, 46 – 79
Peripheral nerve procedure	19, 10 – 29	11, 8 – 15	15, 10 – 20	47, 31 – 62
Stereotactic radiosurgery	2, 0 – 4	3, 1 – 5	6, 2 – 11	12, 5 – 18
Cranioplasty	14, 11 – 17	12, 10 – 14	12, 10 – 14	37, 32 – 44
