# Supplementary material for: Procedures performed during neurosurgery residency in Europe
Source: Acta Neurochir (Wien). 2020 Aug 16;162(10):2303–11. doi: 10.1007/s00701-020-04513-4 (PMC7496021; doi:10.1007/s00701-020-04513-4)
Supplement: Supplementary file 3 — (PDF 26 kb) [file 701_2020_4513_MOESM3_ESM.pdf]

Supplemental Table 3: Basic demographic information of the survey responders, comparing the preliminary (n=80) and the new cohort. SD = standard deviation.

**Variable**	**Preliminary cohort**	**New cohort**	**p-value**
Age in years (mean ± SD)	43.0 ± 8.6	42.4 ± 9.1	0.657
Gender			
Male	71 (88.8%)	77 (87.5%)	0.803
Female	9 (11.2%)	11 (12.5%)	
Type of hospital/employment			
University/teaching hospital	73 (91.3%)	70 (79.6%)	
Other public hospital	5 (6.2%)	16 (18.2%)	0.065
Private hospital	2 (2.5%)	2 (2.3%)	
Country of training			
Albania	- (0%)	1 (1.1%)	
Austria	2 (2.5%)	- (0%)	
Belgium	2 (2.5%)	- (0%)	
Bosnia and Herzegovina	- (0%)	1 (1.1%)	
Croatia	- (0%)	1 (1.1%)	
Czech Republic	- (0%)	1 (1.1%)	
Estonia	1 (1.3%)	- (0%)	
Finland	4 (5.0%)	4 (4.6%)	
France	16 (20.0%)	5 (5.7%)	
Germany	12 (15.0%)	25 (28.4%)	
Greece	1 (1.3%)	11 (12.5%)	
Israel	1 (1.3%)	1 (1.1%)	
Italy	1 (1.3%)	7 (8.0%)	<0.001
Kazakhstan	1 (1.3%)	2 (2.3%)	
Lithuania	1 (1.3%)	- (0%)	
Netherlands	- (0%)	4 (4.6%)	
Norway	7 (8.8%)	- (0%)	
Poland	- (0%)	1 (1.1%)	
Portugal	- (0%)	4 (4.6%)	
Romania	- (0%)	1 (1.1%)	
Russian Federation	- (0%)	2 (2.3%)	
Serbia	7 (8.8%)	2 (2.3%)	
Sweden	4 (5.0%)	1 (1.1%)	
Switzerland	9 (11.3%)	4 (4.6%)	
Turkey	- (0%)	4 (4.6%)	

United Kingdom	11 (13.8%)	3 (3.4%)	
Length of residency (mean ± SD)	6.0 ± 1.0	5.8 ± 0.9	0.148
Year of residency graduation			0.946
1976 – 1980	1 (1.3%)	1 (1.1%)	
1981 – 1985	- (0%)	1 (1.1%)	
1986 – 1990	2 (2.5%)	3 (3.4%)	
1991 – 1995	5 (6.3%)	6 (6.8%)	
1996 – 2000	6 (7.5%)	3 (3.4%)	
2001 – 2005	8 (10.0%)	7 (8.0%)	
2006 – 2010	12 (15.0%)	16 (18.2%)	
2011 – 2015	20 (25.0%)	22 (25.0%)	
2016 – 2019	26 (32.5%)	29 (33.0%)	
Part of training in a different country			0.176
No	62 (77.5%)	60 (68.2%)	
Yes	18 (22.5%)	28 (31.8%)	
**Total**	**n=80 (100%)**	**n=88 (100%)**	
