# Supplementary material for: Procedures performed during neurosurgery residency in Europe
Source: Acta Neurochir (Wien). 2020 Aug 16;162(10):2303–11. doi: 10.1007/s00701-020-04513-4 (PMC7496021; doi:10.1007/s00701-020-04513-4)
Supplement: Supplementary file 4 — (PDF 27 kb) [file 701_2020_4513_MOESM4_ESM.pdf]

Supplemental Table 4: Overview on the caseloads of certain types of procedures, performed on average during neurosurgery residency in Europe, comparing the preliminary (n=80)* and the new cohort (n=88)**. CI = confidence interval.

Procedure type	Independent	Supervised	Assisted	Total
	Mean, 95% CI	*Mean, 95% CI*	*Mean, 95% CI*	*Mean, 95% CI*
All procedures	513, 415 – 611* vs. 565, 361 – 770**, p=0.656	520, 366 – 674* vs. 450, 362 – 537**, p=0.414	750, 483 – 1017* vs. 421, 328 – 514**, p=0.018	1805, 1342 – 2269* vs. 1407, 1081 – 1732**, p=0.1557
Cranial procedures	280, 225 – 337* vs. 281, 187 – 376**, p=0.987	266, 204 – 328* vs. 249, 196 – 302**, p=0.665	381, 254 – 507* vs. 239, 181 – 297**, p=0.038	942, 735 – 1148* vs. 767, 586 – 948**, p=0.205
Spinal procedures	205, 149 – 260* vs. 243, 128 – 359**, p=0.561	249, 127 – 371* vs. 192, 146 – 238**, p=0.364	365, 212 – 518* vs. 182, 131 – 233**, p=0.021	838, 532 – 1145* vs. 608, 430 – 787**, p=0.187
Procedures on adult patients	467, 372 – 562* vs. 481, 322 – 641**, p=0.885	478, 341 – 614* vs. 390, 319 – 462**, p=0.246	694, 445 – 943* vs. 375, 290 – 459**, p=0.013	1666, 1248 – 2083* vs. 1230, 966 – 1493**, p=0.075
Procedures on pediatric patients	35, 20 – 50* vs. 61, 5 – 117**, p=0.406	45, 16 – 73* vs. 122, -17 – 261**, p=0.309	79, 21 – 137* vs. 47, 31 – 64**, p=0.276	160, 59 – 262* vs. 237, 69 – 405**, p=0.449
