# Supplementary figures and images for: Procedures performed during neurosurgery residency in Europe
Source: Acta Neurochir (Wien). 2020 Aug 16;162(10):2303–11. doi: 10.1007/s00701-020-04513-4 (PMC7496021; doi:10.1007/s00701-020-04513-4)

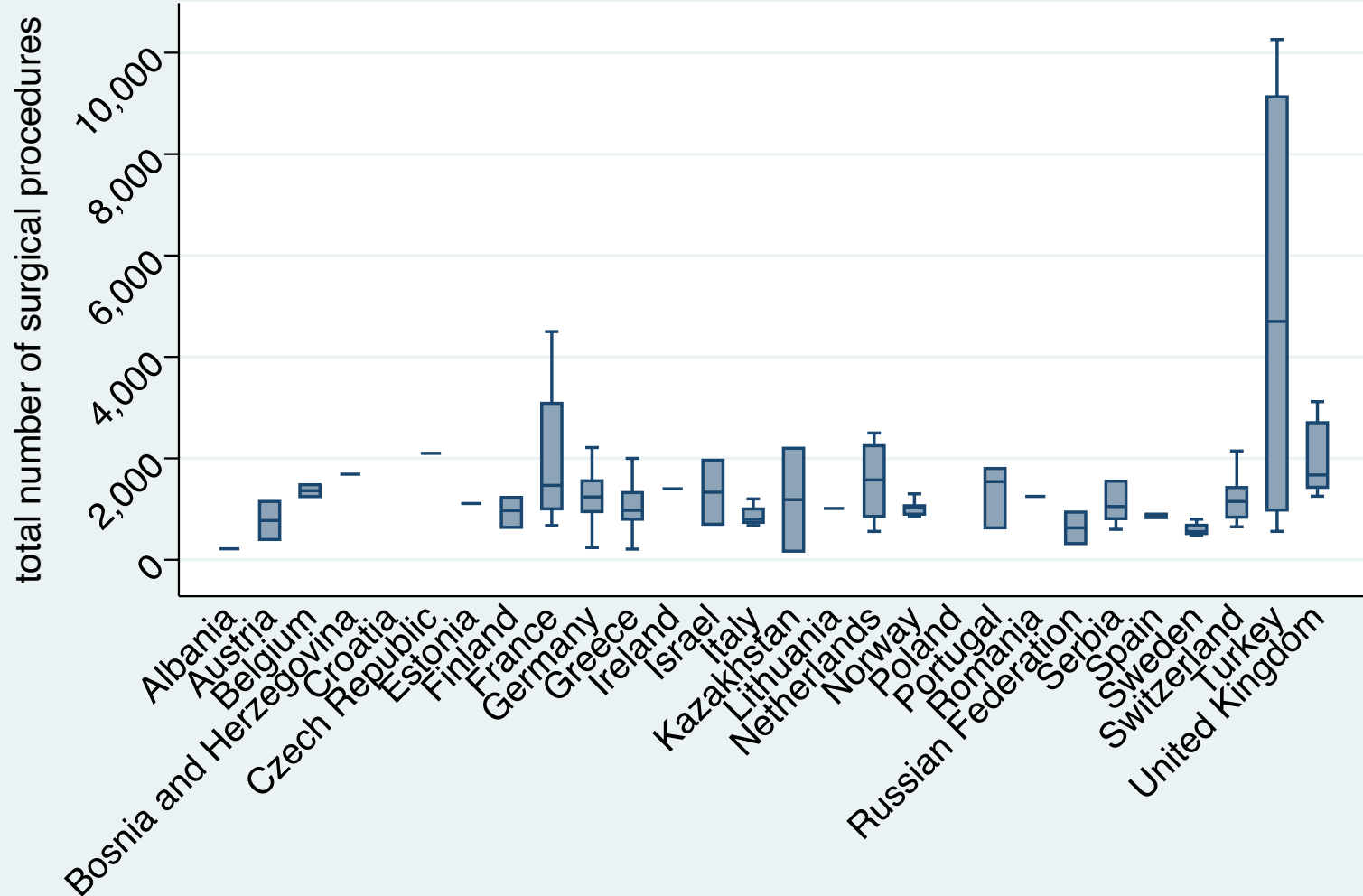

excludes outside values

Supplement: Supplementary file 5 — Box plots of the total number of surgical procedures performed and assisted during residency by trainees from different EANS countries. The figures display the median with the 25th–75th percentile (box), the upper and lower adjacent values (whiskers). Outliers are not visualized. (PDF 18 kb) [file 701_2020_4513_MOESM5_ESM.pdf]
